# Development of a skills-based curriculum to equitably promote health behaviors through physical activity

**DOI:** 10.3389/fpsyg.2025.1559497

**Published:** 2025-11-24

**Authors:** Anna Schwartz, Heather Lewis, Lisa Jo Gagliardi, Scott Martin, Nick Jaskiw, Nancy J. Jaskiw, Rebecca E. Hasson

**Affiliations:** 1 School of Kinesiology, University of Michigan, Ann Arbor, MI, United States; 2 Williamston Community Schools, Williamston, MI, United States; 3 LJ Gagliardi, LLC and Michigan School Health Coordinators’ Association, Hessel, MI, United States; 4 Michigan Department of Education, Lansing, MI, United States; 5 Newaygo County Regional Educational Service Agency, Fremont, MI, United States; 6 Reeths-Puffer Intermediate Schools, Muskegon, MI, United States

**Keywords:** family engagement, curriculum, physical activity, child health, family relationships

## Abstract

**Background:**

A systematic process was used to develop a curriculum that empowers families to engage in health behaviors through the common thread of physical activity.

**Methods:**

A 12-step curriculum integration process was followed: assemble curriculum development team; determine scope; consider level of physical activity integration; consider vertical and horizontal physical activity integration; create module working groups; create learning outcomes; document content; determine themes; determine sequencing; select assessments; communicate with partners; re-evaluate and revise.

**Results:**

A multidisciplinary team created a 12-module family-based curriculum. PA was nested throughout; horizontal and vertical integration were achieved by including PA across topics and modules. Module working groups were based on expertise, with learning outcomes developed. Content focused on improving knowledge, attitudes, and skills, with the theme of families “moving together, thinking together and being together.” Module order aligns with Maslow’s hierarchy of needs. Assessments will include pre/post knowledge, attitude, and skill questions. Key partners reviewed the curriculum, with revisions completed.

**Discussion:**

This family-based curriculum provides a needed resource for families and for potentially fostering school-home connections. The systematic development of a family-based curriculum with physical activity integrated further indicates the potential of using physical activity to promote family engagement in health behaviors.

## 1 Introduction

The COVID-19 pandemic created widespread challenges for children and adolescents, disrupting nearly every aspect of daily life. Families with children experienced increased food insecurity (Ahn and Norwood, 2021; Parekh et al., 2021), and youth faced sleep problems (Becker et al., 2021; Murata et al., 2021; Sliwa et al., 2023), heightened anxiety and depression symptoms (Racine et al., 2021; Madigan et al., 2023), social isolation and loneliness (Farrell et al., 2023; Velez et al., 2022; Jones et al., 2022), and setbacks in academic achievement (Kuhfeld et al., 2020; Abrams et al., 2022; Kuhfeld et al., 2022). Adolescents missed key milestones (Velez et al., 2022), limiting opportunities for personal growth. These impacts were not experienced equally; the pandemic exacerbated existing inequities, particularly among children from lower-income and racially and ethnically diverse backgrounds, who faced greater risks of food insecurity, school closures and associated mental health difficulties, and obesity (Abrams et al., 2022; Ambrose, 2020; Oberg et al., 2022; Parekh et al., 2021; Hawrilenko et al., 2021; Jenssen et al., 2021). School closures further limited access to essential resources such as healthcare, nutrition, and adaptive physical education (Masonbrink and Hurley, 2020; García and Weiss, 2020; Kim et al., 2022), creating new barriers to meeting children’s basic and developmental needs.

Maslow’s hierarchy of needs (Maslow, 1943; McLeod, 2007) provides a useful framework for understanding these challenges. According to this theory, human needs are structured into a hierarchy, where needs at one level must be satisfied before advancing to the next level, and behavior is motivated by these needs. The hierarchy includes the following levels, from bottom (most basic needs) to top (higher-level needs): physiological needs, safety and security needs, love and belonging needs, self-esteem needs, and self-actualization needs. Physiological needs refer to biological necessities for survival such as food, air, water, and shelter (McLeod, 2007). Safety needs involve having stability and control over one’s life, including emotional and financial security, and family stability (McLeod, 2007). Love and belonging needs include having relationships with others and feeling connected, such as having friends, family, and mutual affection (McLeod, 2007). Esteem needs refer to having both self-esteem (dignity) as well as having respect from others, such as feeling valued and having confidence (McLeod, 2007). Finally, self-actualization needs include reaching one’s full potential and focusing on personal growth (McLeod, 2007). The pandemic disrupted children’s lives across all levels of this hierarchy – from physiological (e.g., food insecurity, sleep difficulties) and safety (e.g., mental health concerns) to belonging (e.g., isolation), esteem (e.g., academic achievement), and self-actualization (e.g., missed milestones). Framing the effects of COVID-19 in this way highlights both the breadth of needs that must be addressed and the inequities intensified during this period.

Physical activity can serve as a powerful mechanism to address the full spectrum of youth needs. It promotes physiological health by reducing long-term risks of heart disease, type 2 diabetes, and some cancers (Physical Activity Guidelines Advisory Committee, 2018), supporting basic health needs. Physical activity also benefits mental health among youth (Biddle and Asare, 2011; Biddle et al., 2019), potentially enhancing feelings of safety and security for young people. Additionally, physical activity can foster social emotional learning (Goh et al., 2022) and contribute to reduced loneliness (Pels and Kleinert, 2016), helping to address love and belonging needs. Academic achievement is also positively impacted (Álvarez-Bueno et al., 2017) - as exercise primes the brain for learning (Hillman et al., 2008, 2009; Szuhany et al., 2015) - supporting self-esteem, while personal fulfillment helps motivate adolescents to be active (Haverly and Davison, 2005), fulfilling self-actualization needs. Despite its benefits, physical activity remains an often overlooked, undervalued, and underutilized resource for meeting children’s needs. In fact, physical activity levels declined during the COVID-19 pandemic (Rossi et al., 2021; Physical Activity Alliance, 2022) and recent reports indicate that less than 30% of U.S. youth ages 6-17 years meet the recommendation of achieving 60-minutes of moderate-to-vigorous physical activity each day (Physical Activity Alliance, 2022). While parents and caregivers sought online information to support their children’s health during the pandemic (Negrone et al., 2023), there was a shortage of resources to promote youth physical activity and address children’s diverse needs across the Maslow hierarchy.

Existing family-based interventions often focus on a single primary outcome – such as treating emotional and behavioral disorders (Kaslow et al., 2012) or improving nutrition (Black et al., 2017). Some family-based interventions extend beyond increasing physical activity alone by incorporating other outcomes like quality time or dietary improvements (Brown et al., 2016). However, there remains a gap in interventions that leverage physical activity to simultaneously support child health and development. Importantly, family-based physical activity interventions also present an opportunity to strengthen family functioning and well-being while meeting children’s needs. For example, in a review, Rhodes et al. (2024) found that these interventions may be able to enhance sub-domains of family functioning, such as cohesion and organization.

Building on this foundation, the present study explicitly connects physical activity to a range of health behaviors while directly fostering family connections and well-being. In addition, it positions physical activity as a strategy to equitably address a wide variety of needs in the wake of the COVID-19 pandemic. Finally, by applying Maslow’s hierarchy of needs as a guiding framework, this study introduces an innovative approach that organizes children’s needs and highlights how physical activity can be used to support them across all levels of the hierarchy. The purpose of this study was to develop a family-based curriculum to achieve three main objectives: (1) provide families with resources to engage in healthy behaviors together at home; (2) empower families to support their child’s needs through physical activity; and (3) ensure that the resources are able to equitably meet the needs of all families. By integrating physical activity throughout the curriculum, it aims to help children meet the physical activity guidelines while also developing other health behaviors, thereby supporting their overall health, well-being and addressing the full hierarchy of needs.

## 2 Methods

### 2.1 Curriculum development

The curriculum development process followed the twelve-step curriculum integration framework proposed by Malik and Malik (2011). Although originally designed for medical school curriculum integration, Costley (2015) suggests that these steps can also be applied for use in secondary and elementary schools, suggesting broader applicability beyond medical education. We are further adapting the steps for the creation of a family-based curriculum. We selected this framework because, unlike more general curriculum development approaches (e.g., Kern’s six steps; Kern, 2016), Malik and Malik’s (2011) framework provides a stepwise guide specifically focused on *integration* of content areas. Since our goal was to intentionally embed physical activity within discussions of other health behaviors (e.g., sleep, nutrition, resilience), we found this approach well-suited to the aims of our project.

In adapting the steps, the curriculum integration process was adjusted to create a curriculum specifically for families rather than medical students. Certain steps were modified to align with the goal of developing a curriculum that integrates physical activity throughout. Instead of integrating two academic subject areas (e.g., basic science and clinical skills) (Malik and Malik, 2011), the focus here was on weaving one health behavior- physical activity- into discussions of other health behaviors. This adjustment is particularly evident in steps 3 and 4.

**Figure 1:**
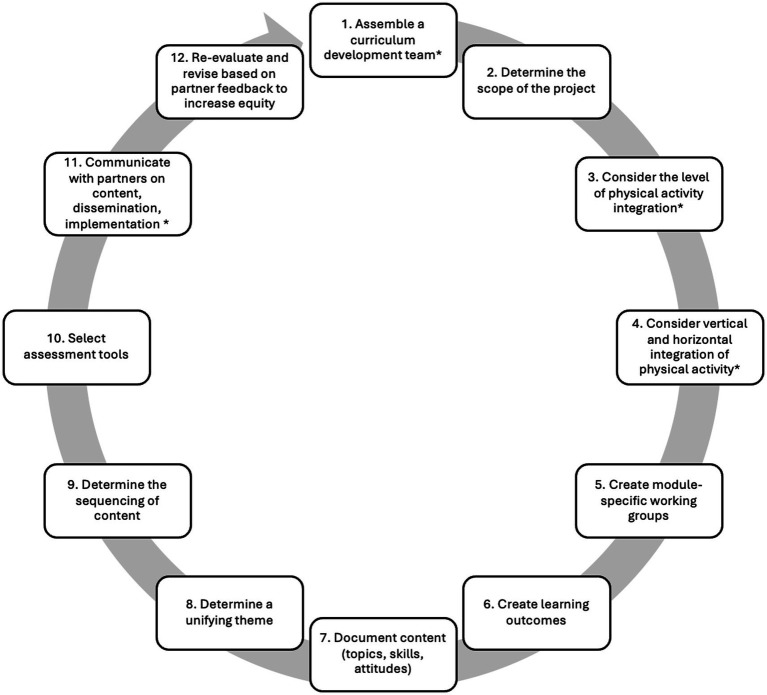
Twelve steps of curriculum integration, based on Malik and Malik (2011). *Indicates steps that were adapted.

Further adaptations included changes to step 1, where “train the staff members” was reframed as assembling an expert team, and step 11, where “communicate with students and staff” was modified to involve communication with expert partners (Malik and Malik, 2011). The twelve steps taken are outlined below and summarized in Figure 1 and Table 1.

**Table 1:** Twelve step curriculum integration process adapted from Malik and Malik (2011).

Steps	Steps (Malik and Malik, 2011)	Methods	Results
1	Train the Staff Members (Adapted)	Assemble a curriculum development team.	A multidisciplinary team was formed consisting of a variety of individuals from multiple levels within the education system as well as outside of it.
2	Decide on scope of integration	Determine the scope of the project.	The team decided to create a 12-module curriculum based on a common vision, mission, and core value rooted in physical activity promotion.
3	Choose the level of integration (Adapted)	Consider the level of physical activity integration into the curriculum to create a common thread, using the curriculum ladder (Harden, 2000).	The integration level of nesting was selected; physical activity was infused into each health-specific module (Harden, 2000).
4	Go for both vertical and horizontal integration (Adapted)	Consider vertical and horizontal integration of physical activity into the curriculum.	Vertical integration was achieved by integrating physical activity into each module (intended to be used at different times) and horizontal integration was achieved by integrating physical activity into all materials related to an individual module.
5	Establish working groups and elucidate their responsibilities	Create module-specific working groups based on expertise of curriculum development team members.	Module-specific working groups were created with one or more team members contributing to each module. Each module underwent team review, feedback, and revisions until complete.
6	Determine learning outcomes	Create learning outcomes.	Learning outcomes were created for each module from the finalized curriculum materials, utilizing Generative AI (ChatGPT 4.0/3.5).
7	Identify the contents (knowledge, skills and attitude)	Document content, including topics covered, skills learned, and attitudes developed.	Modules encompassed a variety of health topics, with each featuring a main section with text and images to impact knowledge and attitudes, an infographic for easy reference, and an activity calendar to build skills. Physical activity was integrated throughout.
8	Create Themes	Determine a unifying theme for the curriculum.	The toolkit’s theme, “Move together, think together, and be together,” was designed to help families be active together, problem-solve together, and strengthen family bonds across health topics.
9	Prepare a comprehensive timetable	Determine the sequencing of curriculum content.	The twelve modules comprising the family engagement toolkit were organized in a 12-month sequence aligned with Maslow’s hierarchy of needs (Maslow, 1943), allowing families to progress from meeting physiological needs to self-actualization.
10	Select assessment methods	Select appropriate assessment tools.	Questions assessing knowledge, attitudes, and skills will be included at the beginning and end of each module to assess pre/post changes.
11	Communicate with students and staff (Adapted)	Communicate with partners on content, dissemination, and implementation.	The completed modules were distributed to the Michigan Department of Health and Human Services (MDHHS), MDHHS Success Coaches, the Michigan School Nurses’ Association, and the Michigan School Health Coordinators’ Association for expert review.
12	Commit to re-evaluation and revision	Re-evaluate and revise the curriculum based on partner feedback to increase equity.	Revisions were made based on key partner feedback and internal review, with an emphasis on equity, including revising the modules to be at an eighth grade reading level and including infographics. The curriculum development team committed to an ongoing process of review and revision.

#### 2.1.1 Step one: assemble a curriculum development team

A school district consultant from the Michigan Department of Education (S.M.) assembled a team of individuals with the necessary background and expertise to contribute to this project. Individuals with knowledge of a variety of health areas that promote academic, behavioral, social, and emotional learning were considered so that a diverse curriculum could be created covering a comprehensive set of health topics that are important to child health. Team members joined with knowledge of the project and the plan to create an integrated curriculum with topics and learning outcomes that built upon each other in a progressive manner. The team planned to co-create all aspects of the curriculum and thus did not need to be familiarized with any pre-established processes or expectations.

#### 2.1.2 Step two: determine the scope of the project

The curriculum development team met via teleconferencing meetings to determine the scope of the project. In joining the project, all team members shared a common goal of creating a family engagement curriculum that promotes physical activity. This goal was refined through discussion to develop a common vision, mission, and core value to guide the development of the curriculum.

The structure of the curriculum and process for development was also discussed. Key components of a curriculum were considered such as objectives (i.e., anticipated outcomes), content and subjects to cover (i.e., what material is taught), and learning experiences (i.e., how the material is learned) (Lunenburg, 2011).

#### 2.1.3 Step three: consider the level of physical activity integration

In this project, integration was conceptualized as the inclusion of physical activity within discussion of other health topics, rather than integrating two specific subject matters. The curriculum development team sought to create a common thread of physical activity through every section of the curriculum - “integrating” physical activity into various health topics. The team wanted to create a comprehensive health curriculum for families that honored their diverse needs.

The extent to which physical activity would be integrated was identified in Harden (2000)’s “curriculum integration ladder.” This ladder was developed for integration within medical education and discussed by Malik and Malik (2011). The ladder contains eleven different “rungs” of integration, with each step up on the ladder indicating greater integration. It ranges from “isolation,” where subjects are taught completely independent of one another with no connections made, to “trans-disciplinary,” where integration is done by an individual in real-world situations (Harden, 2000).

#### 2.1.4 Step four: consider vertical and horizontal integration of physical activity into the curriculum

The curriculum development team considered vertical and horizontal integration of physical activity. Malik and Malik (2011) describe vertical integration as integration between topics taught at different times or phases in a curriculum, while horizontal integration refers to integration between topics taught at the same time or phase in the curriculum. Thus, the curriculum development team considered the potential structure of the curriculum and determined if vertical, horizontal, or both types of integration were necessary to fully integrate physical activity.

#### 2.1.5 Step five: create module-specific working groups

Working groups were created to divide the creation of the curriculum among all team members. Each group was tasked with developing materials related to a specific topic. The expertise of each team member was considered to ensure individuals were responsible for creating content that most aligned with their area of practice. In some cases, multiple team members worked on content for the same topic, while in other cases, only one team member created content for a given topic.

#### 2.1.6 Step six: create learning outcomes

The desired learning outcomes were considered when developing the materials, with outcomes ultimately compiled and refined at the end of the curriculum development process.

#### 2.1.7 Step seven: document content (topics covered, skills learned, attitudes developed)

Prior to writing a section of the curriculum, the curriculum development team members meticulously planned out the given content. Knowledge to be gained, skills to be learned, and desired attitudes to be conveyed were all considered to ensure a clear structure for drafting the curriculum and later refinement.

#### 2.1.8 Step eight: determine themes

As indicated by Malik and Malik (2011), themes help elucidate the “big picture” of a curriculum. The curriculum development team sought to create a unifying theme for the curriculum that could be woven throughout the content. This was developed through ongoing discussions between the team members.

#### 2.1.9 Step nine: determine sequencing

The order in which the information in the curriculum was presented needed to be clear, with Malik and Malik (2011) indicating that there should be a flow between material and that it should be presented in a logical order. Once the curriculum was complete, the curriculum development team reviewed all content created and determined how to sequence it to help families achieve the greatest benefit. Two major considerations were: (1) how to best scaffold learning so that the skills learned in the first content sections were used and built upon in the later parts of the curriculum, and (2) alignment with the U.S. Kindergarten through 12th grade academic year.

#### 2.1.10 Step ten: select assessment methods

The curriculum was designed to ultimately be disseminated across the state of Michigan to diverse families living in urban, rural, and suburban contexts. The curriculum development team thus selected appropriate assessment methods tailored for practice-based settings and wide dissemination. They ensured that the methods would effectively gauge parent knowledge, attitudes, and skills related to the modules. Though the modules are intended to engage the entire family, the team discussed focusing assessments on changes in parents, as they are the key facilitators of the modules, and their behaviors can influence those of their children. For example, parental role-modeling and support are key for child physical activity (Hutchens and Lee, 2018; Yao and Rhodes, 2015).

#### 2.1.11 Step eleven: communicate with partners on content and implementation

The curriculum development team engaged school partners in Michigan to gather feedback on the created curriculum and develop implementation and dissemination strategies for parents. These partners, who have significant experience in school-home interactions, provided valuable insights on content and delivery. This step was adapted from the Malik and Malik (2011) steps to specifically incorporate feedback from school partners.

In May 2024, the curriculum was presented to the Michigan School Health Coordinators Association (MiSHCA). The presentation included an overview of the curriculum and case studies illustrating how various modules could address diverse child needs. For instance, one case study described an intergenerational family with varying physical activity preferences and engagement levels seeking to strengthen family connections. The Love and Belonging modules were reviewed as potential resources for such families. At the end of the session, attendees were asked to rate the curriculum’s value to the association on a scale of 1 to 5, with five indicating greater value.

#### 2.1.12 Step twelve: re-evaluate and revise curriculum based on partner feedback

Revisions were made based on feedback from partners and further revisions were made following internal review by the curriculum development team. The entire curriculum was carefully examined to ensure it provided fair and inclusive opportunities for all families, regardless of their background, abilities, or circumstances. This process involved assessing whether the curriculum content, teaching methods, materials, and assessment practices were designed to accommodate and support the diverse needs of children. Additionally, the environment and circumstances under which the curriculum would be implemented were evaluated, considering factors such as the cultural and socioeconomic background of families, and any potential barriers to equitable access to engagement. Any identified inequities or barriers were addressed by making appropriate changes to the curriculum or its implementation, such as modifying content, providing additional resources or support, or adapting teaching methods.

## 3 Results

The outcome of each step is detailed below as well as in Table 1.

### 3.1 Step one: assemble a curriculum development team

Table 2 describes the curriculum development team, with expertise to create content covering a wide range of health topics. The team included a diverse set of experts from multiple levels of the school system as well as from outside of the school system.

**Table 2:** Overview of the 12-module family-based curriculum.

Maslow’s hierarchy	Module title	Learning outcomes	Team member(s)	Team member expertise
Foundation	Family discussions	1. Understand the value of meaningful discussion. 2. Establish collaborative communication guidelines. 3. Practice effective communication techniques. 4. Use physical activity to regulate emotions and improve family discussions.	Nathan Maynard	Behavioral management expert; co-author of “Hacking School Discipline” and co-founder of behavior flip, a restorative behavior management software; degree in neuroscience.
Physiological	Healthy choices	1. Understand the impact of choices on health and well-being. 2. Develop a personalized family action plan for physical activity. 3. Motivate healthy behaviors through knowledge and community. 4. Promote physical activity as a choice to improve family health.	Evilia Jankowski	Michigan’s State School Nurse Consultant; formerly collaborated with the CDC and American Academy of Pediatrics.
	Rebecca Hasson	Pediatric Exercise Physiologist; Associate Professor of Movement Science at the University of Michigan, with expertise in exercise physiology, implementation science, and health equity research.
	Nutrition	1. Understand the role of nutrition and energy balance. 2. Identify strategies for healthy eating and snacking. 3. Recognize the connection between nutrition and physical and mental health. 4. Understand how balanced nutrition and physical activity support energy levels and overall health.	Natalie Queen	Physical education teacher; master’s degree in school guidance counseling and degrees in emotional impairment, family & consumer sciences, and physical education.
Sleep	1. Understand the role of sleep in overall health. 2. Identify strategies for improving sleep quality. 3. Recognize the connection between daily activities and sleep. 4. Recognize how physical activity enhances sleep quality and health.	Nancy Jaskiw	School psychologist; prior research on infant sleep disorders; provides psychological services to her local school community and workshops for the broader community.
Safety	Schedules and routines	1. Understand the value of schedules and routines. 2. Develop and adapt family schedules. 3. Foster positive habits through routines. 4. Learn how to include physical activity in family routines to enhance health and bonding.	Scott Martin	Michigan Department of Education Consultant; former teacher, principal, and coach; advocates for supports to meet the needs of students across Michigan.
Love and belonging	Family team building	1. Understand the role of family as a team. 2. Strengthen family bonds through activities. 3. Develop strategies for effective family teamwork. 4. Understand how family teamwork and physical activities strengthen relationships and promote healthy habits.	Nick Jaskiw	School psychologist (region); established the Intensive Student Support Network in a public school system and contributes to state and regional initiatives.
	Lifelong skills	1. Identify and support lifelong skill development. 2. Recognize the role of physical activity in emotional regulation. 3. Implement family strategies for skill building. 4. Learn how physical activity helps develop lifelong skills.	Penelope Friday	Former State Childhood Obesity Prevention Coordinator in Indiana; experience with school and early childhood wellness initiatives; doctoral student at the University of Michigan School of Kinesiology.
			Heather Lewis	K-12 Social emotional and mindfulness coordinator; master’s degrees in psychology, in education, and in child development; specializes in social–emotional learning, mindfulness, and student athlete mental health.
Esteem	Feeling good	1. Identify and define well-being. 2. Apply strategies to improve well-being. 3. Develop a plan for family engagement. 4. Demonstrate how physical activity boosts well-being and health.	Evilia Jankowski	See above.
	Substance use and your body	1. Identify protective and risk factors. 2. Explain the role of physical activity in substance misuse prevention. 3. Demonstrate refusal skills and prevention strategies. 4. Encourage regular physical activity to prevent substance misuse and support mental well-being.	Christina Holmes	Regional school health coordinator; certified prevention specialist; former middle school and high school teacher.
			Kelly Johnson-Sager	Regional school health coordinator; certified prevention specialist; coordinates grant programs on prevention, social–emotional learning, and mental health.
	Focus	1. Understand the importance of focus for families. 2. Apply strategies to enhance focus. 3. Encourage family-based activities to build focus skills. 4. Learn how physical activity enhances focus and concentration for children and adults.	Lisa Jo Gagliardi	Whole child consultant; child and adolescent health consultant, facilitator and coach; founder of LJ Gagliardi, LLC, with work focused on child health, social–emotional wellness, and family empowerment.
Self-actualization	Personal best	1. Understand the concept of personal best and its process-focused nature. 2. Develop a growth mindset and apply it to goal setting. 3. Set and achieve personal goals with family support. 4. Understand how goal-setting and physical activity help achieve personal best through a growth mindset.	Alanna Price	Adaptive physical education teacher in a local public school system.
			Rebecca Hasson	See above.
			Lisa Jo Gagliardi	See above.
			Heather Lewis	See above.
			Scott Martin	See above.
			Nick Jaskiw	See above.
			Nancy Jaskiw	See above.
	Resilience	1. Define resilience and the resilient zone. 2. Implement techniques for enhancing resilience. 3. Cultivate positive relationships and mindful habits. 4. Use physical activities to maintain emotional resilience and stay in the “resilient zone” during stress.	Heather Lewis	See above.

### 3.2 Step two: determine the scope of the project

The curriculum development team met biweekly for approximately two years and established a common vision, mission, and core value for the project, included in Table 3.

**Table 3:** Vision, mission, and core value of the curriculum development team.

Team Philosophy	Statement
Collective vision	The Interrupting Prolonged sitting with ACTivity (InPACT) at Home family toolkit is the premier resource in the state of Michigan to strengthen school-home connections around children’s health, wellness, and achievement.
Mission	To empower families to move together, think together, and be together, and to practice health behaviors through the common thread of physical activity.
Core value	We believe engaging in regular physical activity is one of the most effective ways to promote mental and physical health, well-being, and achievement for children, families, and communities.

Statements are from the Interrupting Prolonged sitting with ACTivity (InPACT) at Home Family Toolkit, available at inpactathome.umich.edu and inpact.kines.umich.edu

With these common guiding statements, the team decided that the curriculum would consist of 12 modules, with each module offering information on a new health topic and materials to engage families. Twelve modules were created to allow for there to be one module a month for families to work through; the modules together comprise the full family curriculum. Each module was planned to include a main educational portion, a 20-day activity challenge for families to complete, and supplemental materials as necessary. The educational portion was designed to enhance knowledge and attitudes of a health topic. The activity challenge was designed to help families practice skills taught in the module [e.g., helping families develop Specific, Measurable, Attainable, Relevant, and Timely (SMART) goals]. Finally, the inclusion of supplementary materials varied by module. For example, a progressive muscle relaxation script was included in the Sleep Module that parents could use to help their child develop healthy sleep behaviors (i.e., skill development).

### 3.3 Step three: consider the level of physical activity integration

To integrate physical activity throughout each module, the “nesting” level of integration was selected, which is level four of eleven on the curriculum integration ladder (Harden, 2000). Nesting indicates that content from one subject may be infused into another subject to improve the teaching of it; topics are still taught in a subject-specific manner (Harden, 2000; Fogarty, 1991). The authors created modules focusing on one health topic (e.g., nutrition) but infused physical activity throughout to support and enhance the learning of the main topic in the module. This level of integration was applied to all twelve modules.

### 3.4 Step four: consider vertical and horizontal integration of physical activity into the curriculum

The curriculum development team included both vertical and horizontal integration of physical activity. One module was intended to be used per month in the toolkit. Vertical integration involved integrating physical activity into all modules to create coherence across topics taught at different times. Horizontal integration involved infusing physical activity throughout materials related to each individual module to create cohesion among information used simultaneously. Overall, physical activity was embedded across all modules (vertical integration) and throughout each module and its respective materials individually (horizontal integration).

### 3.5 Step five: create module-specific working groups

The curriculum development team allocated module creation based on individual expertise. The topic of each module and respective contributors from the curriculum development team are shown in Table 2. After each module was drafted by the expert(s) in the topic area, the module was reviewed by the entire curriculum development team, feedback was provided, and revisions were made. This process was continued until each module was considered complete.

### 3.6 Step six: create learning outcomes

Once the modules were created, learning outcomes were developed related to the topic matter in each module (see Table 2). Specific outcomes were drawn from the finalized curriculum materials utilizing generative Artificial Intelligence (ChatGPT 3.5/4.0) to provide families with clear learning goals for each module and guide them in achieving meaningful takeaways.

### 3.7 Step seven: document content (topics covered, skills learned, attitudes developed)

Modules covered twelve different topics (see Table 2). Each module contained a main educational portion with text and images to improve the knowledge and attitudes around a given topic. This main portion was centered around helping families connect a given topic to physical activity, think through the relevant components of a health topic as a family, and find ways to be together while engaging in healthy behaviors. Throughout each module, additional resources were provided for families, including videos made by the curriculum development team and links to reputable resources for further information.

Each module included an activity calendar to help families build the skills to engage in the given health behavior. Activity calendars included a month of activities (4 weeks, 5–6 days/week) so that families could engage in a variety of activities designed to help them practice what they learned in the module and solidify the new behavior. Physical activity was integrated into the calendars by providing families with links to high quality, 8-min physical activity videos from an evidence-informed physical activity program to complete as a family each day (Hasson et al., 2022; Beemer et al., 2023, 2025) (see inpactathome.umich.edu) or other ways to be active as a family. Further, the calendars were designed to be approximately 20 days to give families the flexibility of completing activities during any 5 days of the week; a 20 day calendar has also been used in previous home-based physical activity interventions (Beemer et al., 2025). Research additionally indicates that 20 days is within the time-frame that it may take a habit to form (Lally et al., 2010).

Finally, for select modules, supplementary materials were included to further enhance skill building or knowledge of materials. For example, for the Family Discussions module, a page of over 100 conversation starters was included to help families practice their discussion skills.

Physical activity was integrated throughout the components of each module. In the educational portion, connections to physical activity were made in each module, and physical activity opportunities were included as part of the activity calendars.

### 3.8 Step eight: determine themes

To emphasize the common thread of physical activity, one coordinating theme was created for all the modules, derived from the mission of the group: “Move together, think together, and be together.” “Move together” refers to families being physically active together, “think together” refers to families problem-solving together, and “be together” refers to creating a strong family team. The content in each module is centered around these areas to help families apply this theme to each health topic (except the Family Discussions module, which serves to provide foundational material). For example, the Sleep module addresses “move together” by detailing how exercise can aid with sleep and encouraging families to be active together during the day. The module addresses “think together” by encouraging families to think together about what could impact sleep, including nutrition and the use of electronics. Finally, the module addresses “be together” by suggesting the creation of a sleep routine which could involve activities such as reading a book or listening to music together.

### 3.9 Step nine: determine sequencing

The curriculum team organized the module sequence to follow the five levels of Maslow’s Hierarchy of Needs (Maslow, 1943; McLeod, 2007). Additionally, a foundational module was added to help families develop essential communication skills needed to progress through the hierarchy (i.e., the Family Discussions module).

**Figure 2:**
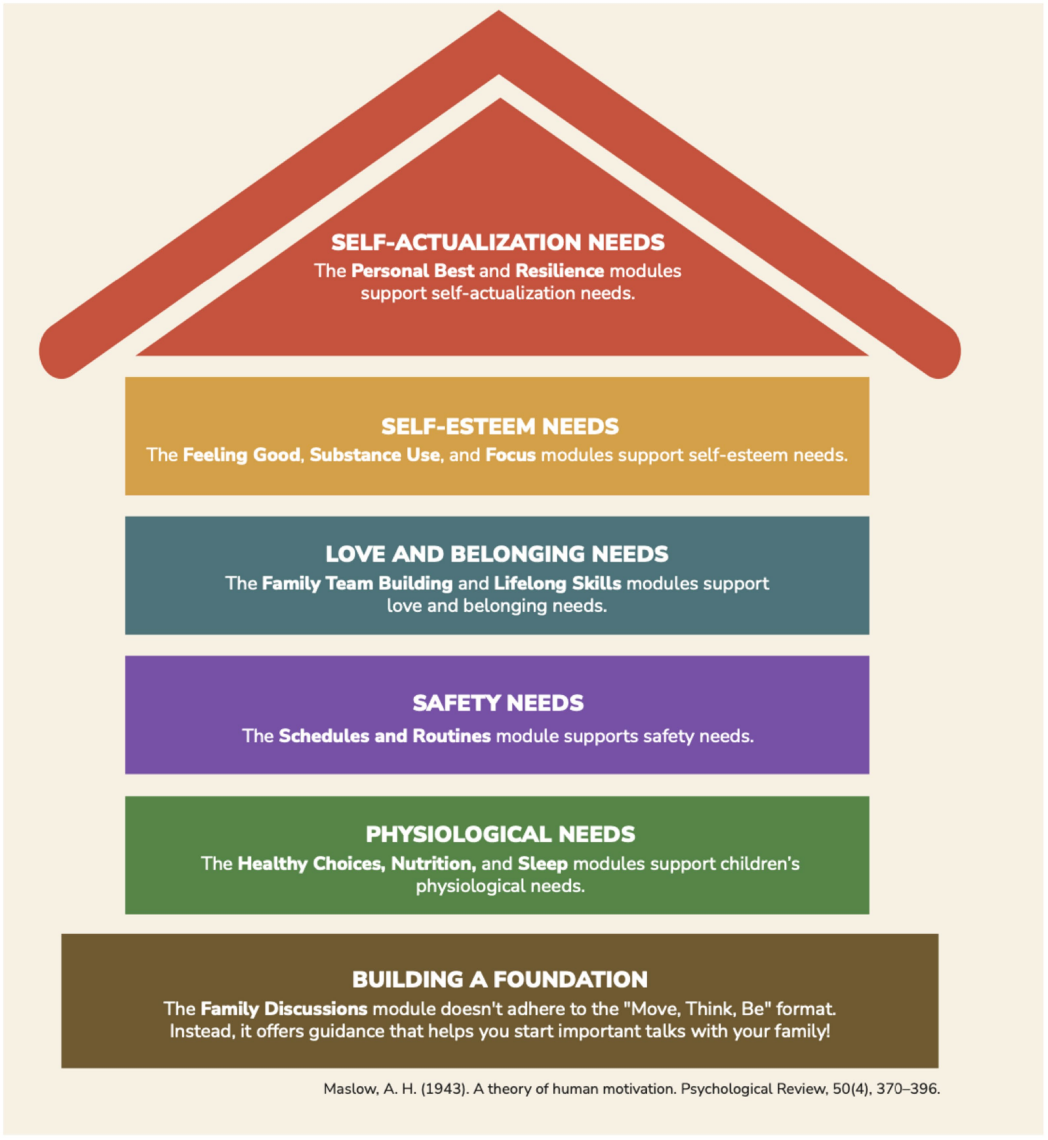
Alignment of the modules with Maslow’s hierarchy of needs (Maslow, 1943). This image is from the Interrupting Prolonged sitting with ACTivity (InPACT) at Home Family Toolkit, available at inpact.kines.umich.edu and inpactathome.umich.edu.

The sequencing of the modules was determined so that each level of the hierarchy could be addressed before advancing to the next, with an ideal pace of working through one module each month of the year. The alignment of the modules with the hierarchy of needs is shown in Table 2 and Figure 2. To align with the standard U.S. Kindergarten through 12th grade academic calendar, families could start with the Family Discussions module in August and progress to the Resilience module by July. Or, module topics can be aligned with relevant times of the year, for example focusing on Sleep in August when children are going back to school or Schedules and Routines in June when children are starting summer break.

### 3.10 Step ten: select assessment methods

The curriculum development team plans to create a survey for each module to help assess changes in knowledge, attitudes, and confidence in their skills to engage in health behaviors. Families will be asked to respond to questions before each module and following the completion of each module to assess changes in these areas. For example, for the Sleep module, families will be asked, “Please rate your level of agreement with the following statement: I have an understanding of the benefits of physical activity for sleep” and “I have strategies that can help me establish a sleep routine for my family.” They will be asked to rate each statement on a scale of 1 (strongly disagree) to 5 (strongly agree) prior to and after completing the module. Pre/post changes in scores could then be assessed to indicate the effectiveness of the module (i.e., show improvements in knowledge, attitudes, and skills). This assessment method is still being developed and has not been implemented at the time of this publication.

### 3.11 Step eleven: communicate with partners on content and implementation

Completed modules were distributed to several partner organizations via teleconferencing presentations, including the Michigan Department of Health and Human Services (MDHHS), MDHHS Success coaches, and the Michigan School Nurses’ Association, and the modules were presented to MiSHCA via a 90-min in-person professional development training. These organizations represent diverse and extensive expertise regarding interacting with parents and thus were able to recommend modifications to the toolkit as needed.

Data was collected following the session delivered to MiSHCA. Twenty MiSHCA members (19 regional school health coordinators, 1 associate member) rated the value of the family curriculum to the organization overall. The overall mean score was 4.65 out of 5, with 5 indicating the greatest value (65% of attendees rated it 5 out of 5 and 35% rated it 4 out of 5).

### 3.12 Step twelve: re-evaluate and revise the curriculum based on partner feedback to increase equity

Revisions were made based on recommendations from key partners and the curriculum development team’s internal review. These changes included adding infographics to visually summarize each health topic, providing an easy reference for parents who may prefer not to read the full module text. The infographic elements were designed using principles from the Health Belief Model (Champion and Skinner, 2008) to encourage family engagement in healthy behaviors. Additionally, references to COVID-19 were removed to ensure the modules remained relevant beyond the pandemic.

Another set of revisions involved adjusting the formatting of the curriculum. The curriculum was reformatted by a professional graphic designer and reviewed by a copyeditor to enhance the readability and consistency of the materials. Infographics were additionally reformatted to fit a consistent color scheme.

Finally, a series of revisions was completed to make the curriculum more equitable. The modules were re-written at an eighth grade reading level using Generative AI (ChatGPT) to ensure that all families were able to use the material. A baseline survey was also created to allow families to assess their needs and use the toolkit in the way that best suits the needs of their family. For example, some families may find they need to focus on basic needs such as nutrition and sleep, whereas others may find it most fitting to start at a different part of Maslow’s hierarchy. The survey helps families tailor the curriculum to their unique circumstances. For instance, if families do not have access to safe spaces in their neighborhoods or communities, they are encouraged to prioritize indoor activities using the evidence-informed physical activity program videos (Beemer et al., 2023, 2025). Next, language and graphics were modified to be body size inclusive throughout the curriculum. Finally, each module was revised to include an audio recording of the material for individuals who prefer to listen to the modules. A future goal is to have each module translated into Spanish and Arabic, which are the two most common languages in Michigan besides English.

Author(s) of each module reviewed their module after recommended revisions were made to ensure compatibility with their initial conceptualization of the module. The curriculum development team further committed to an ongoing process of review and adaptation to accommodate the evolving needs of youth and increase equity and inclusion.

## 4 Discussion

This study aimed to develop a family-based curriculum that integrates physical activity into various health topics, providing families with a resource to engage in healthy behaviors together at home. The resulting 12-module curriculum is designed to empower families to meet their children’s needs using physical activity as a common mechanism. A systematic process was used by following Malik and Malik (2011)’s 12-steps for developing an integrated curriculum. Content was further aligned with Maslow’s hierarchy of needs and revised to address the diverse needs of families. Preliminary data suggests that the curriculum is of high value among key stakeholders.

The toolkit is uniquely designed to address the public health issue of low physical activity by promoting family-based activity. A review by Brown et al. (2016) found that 66% of included family-based interventions positively impacted physical activity. The toolkit includes several effective components discussed in this review: education strategies paired with other interventions, goals that extend beyond health and weight loss, and customization to fit each family’s context. Additionally, the 20-day family challenge calendars in the toolkit emphasize shared physical activity, ideally fostering parent support - a key factor in promoting children’s physical activity (Yao and Rhodes, 2015). Future research is needed to assess the toolkit’s effectiveness in increasing youth and family physical activity.

This toolkit could further potentially address concerns about children’s mental and social–emotional health emerging from the pandemic by strengthening family relationships. A review by Meherali et al. (2021) highlighted the negative impact of crises, including COVID-19, on the mental health of children and adolescents, such as stress, anxiety, and depression. Similarly, qualitative interviews by Watts and Pattnaik (2023) found that the pandemic affected the social–emotional development of children (ages 4–5 years), with impacts including social deprivation, emotional issues, greater externalizing behaviors (e.g., tantrums, hitting), and difficulties achieving life skills. Families and parents could play an important role in addressing these issues.

Strong parent–child relationships were linked to improved child well-being during the pandemic, with stronger connections associated with reduced anxiety and depression and increased happiness (McArthur et al., 2023). Similarly, beyond the pandemic, parent-family connectedness (e.g., closeness and satisfaction with relationships) is a critical protective factor for many adolescent risk behaviors, including emotional distress, suicidality, substance use, and violence (Resnick et al., 1997). Thus, by promoting family activities, the toolkit could help address pandemic-related challenges as well as foster stronger parent–child bonds to benefit child well-being moving forward. Nevertheless, future research is needed to evaluate the toolkit’s effectiveness in strengthening parent–child relationships.

### 4.1 Fostering school-home connections

**Figure 3:**
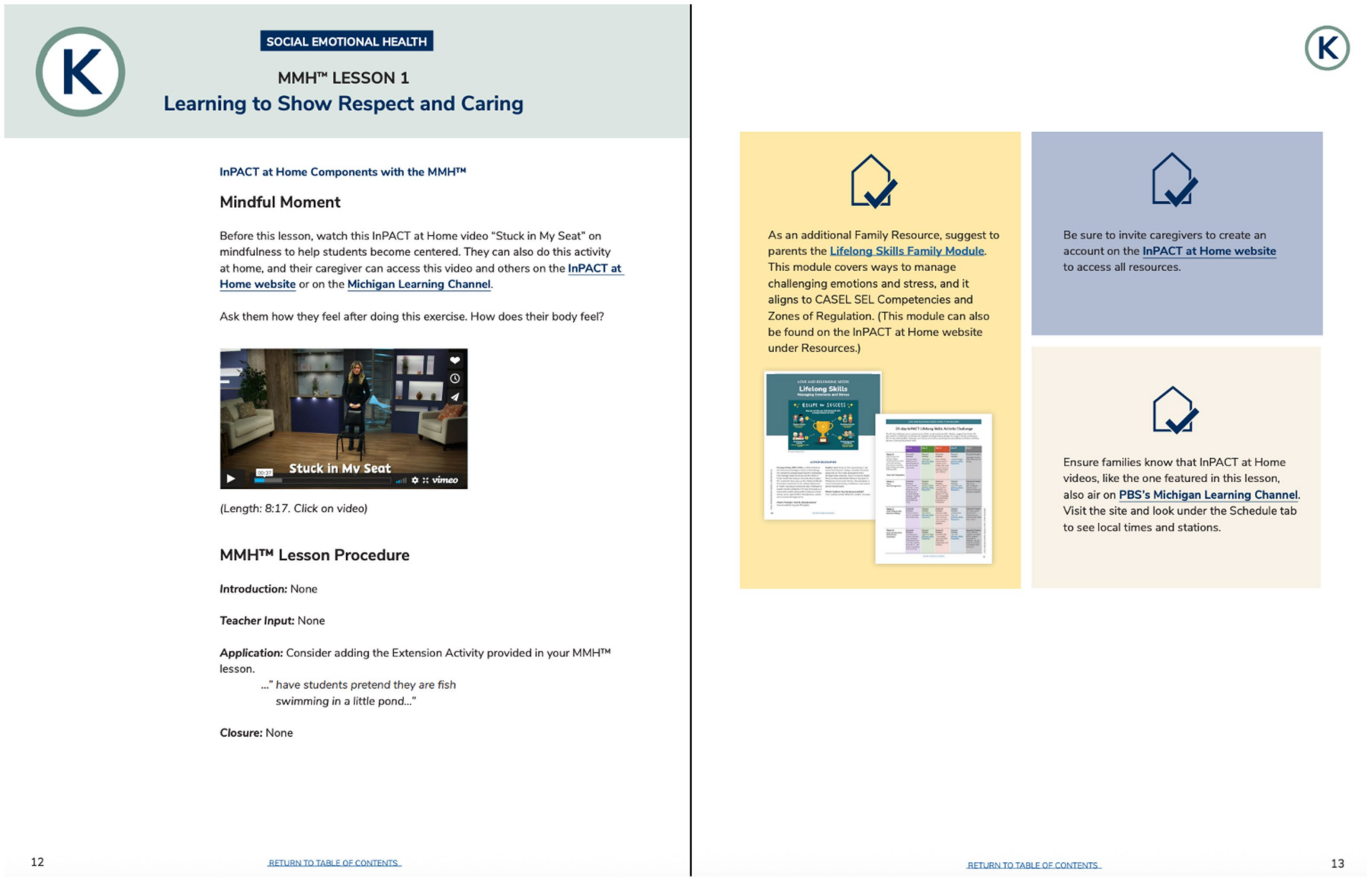
Integration of the Lifelong Skills Module with the Kindergarten Social Emotional unit in the Michigan Model for Health™ (MMH) curriculum. The full integration guide is available at inpact.kines.umich.edu.

During the pandemic, parents looked online for health resources (Negrone et al., 2023; Hartling et al., 2024), indicating there is a potential opportunity for schools to connect with families by providing this information. The toolkit is designed to support families and be utilized by schools, as it is well-suited to strengthen school-home connections in two key ways. First, the toolkit was intentionally designed to complement existing school-based resources. This was accomplished by engaging individuals working at various levels of the education system as members of the curriculum development team. Second, the modules and calendars have been integrated into the state health curriculum, Michigan Model for Health™ (MMH) (Schwartz et al., 2025), as part of a larger effort to provide greater physical activity opportunities and parent materials throughout the curriculum. For Kindergarten through fifth grade, there is a companion integration guide that recommends a module and calendar to send home to parents to complement what is being taught in MMH. For example, for the Kindergarten Social and Emotional Health Unit, it is recommended to send families the Lifelong Skills Module, which provides information on using physical activity to manage emotions (see Figure 3). This integration resembles Duncan et al. (2011, 2019)‘s “Healthy Homework” program, which increased step counts through physical activity and nutrition assignments that encouraged family and parent involvement. While Duncan et al. (2011, 2019) ‘s program involved compulsory homework - whereas the family modules and calendars are an optional, non-mandatory integration into MMH - these findings highlight the value of resources that encourage family participation for increasing physical activity. Moreover, though the integration also contained other elements, the modules and calendars could help support family engagement with the school health curriculum.

As demonstrated by the Michigan Department of Education’s Family Engagement Framework, (MiFamily: Michigan Family Engagement Framework, 2020) and prior research (Michael et al., 2015), family engagement with schools is significant for children’s success. Parental involvement is linked to positive educational outcomes across grade-levels, including grades, attendance, and behavior, and the school-home connection is important for child health (MiFamily: Michigan Family Engagement Framework, 2020; Michael et al., 2015). In terms of health, Albright and Weissberg (2010) highlights the importance of the partnership between parents and schools for social–emotional learning, and a review by van Sluijs et al. (2007) indicates that school-based interventions with a family/community component could increase youth physical activity. Moreover, parents are eager to learn more about the social–emotional curriculum their children are learning in school (Drew et al., 2024), supporting that family engagement through health-based materials could be successful. Thus, the modules in the toolkit could enhance parents’ involvement while supporting critical parent–child connections and physical activity. Regardless, future research is needed to confirm the effectiveness of the toolkit to enhance family engagement with schools.

### 4.2 Universal application

This study provides strategies generalizable beyond the creation of family-based curricula. First, the present study demonstrates the use of the Malik and Malik (2011) 12-step approach for curriculum integration. Though Costley (2015) highlights the purpose and importance of integrated curricula, the process for accomplishing this integration may be less clear. By applying the Malik and Malik (2011) approach and explicitly showing the actions taken to fulfill each step, how steps were adapted to fit the specific needs of the curriculum, and the outcomes for each step, this study provides a helpful example for the creation of integrated curricula that could be applied in various disciplines. Second, the present curriculum was aligned with Maslow’s hierarchy of needs, which provides a universally relevant lens for organizing children’s holistic needs. Though Maslow’s hierarchy may not fully explain human motivation and behavior, it remains a well-known and practical framework for organizing child and adolescent needs and intervention components (Remedios, 2025). This framework could be useful for other interventions and curricula as well and encourage recognition of the multitude of needs children and families may experience.

Although the current family toolkit was developed for families in Michigan and reflects the state’s education system, its principles, development process, and curriculum structure can be adapted to diverse geographic, cultural, and educational contexts. The curriculum development steps—such as assembling an expert team, integrating physical activity across topics, and incorporating partner feedback—are flexible and could be replicated in school districts across the U.S. This is particularly relevant in communities where families have limited access to resources that support child well-being (e.g., school counselors, psychologists, health insurance, or internet access) (Showalter et al., 2023), or where recreation opportunities, safety concerns, and challenges in the built environment limit physical activity (Umstattd Meyer et al., 2016; Lopez and Hynes, 2006; Molnar et al., 2004; Duck et al., 2020).

Beyond the U.S., the framework could be adapted for low- and middle-income countries, where meeting basic physiological and safety needs—such as food security and protection from environmental exposures—may take priority (Cardell et al., 2024; Durao et al., 2020; Burroughs Peña and Rollins, 2017). In higher-income settings, a family-based curriculum could potentially be tailored to reinforce school-based physical activity interventions (Porter et al., 2024). Regardless of the setting, the curriculum development process and curriculum could be modified to reflect social and cultural factors that influence participation (Resnicow et al., 1999).

### 4.3 Strengths and limitations

The present study has several strengths. A multidisciplinary team was formed to create the curriculum, with each module authored by an expert, and the overall curriculum development process was informed by a step-by-step guide for integration (Malik and Malik, 2011). The team’s expertise in the education system ensured the curriculum can support families while complementing school-based resources. Further, an equity lens guided both the curriculum development (e.g., alignment with Maslow’s hierarchy of needs) and revision of the curriculum (e.g., audio recording, visuals), allowing it to address families’ unique needs. Finally, feedback from key partners, who will assist in dissemination, has already been sought and incorporated.

Several limitations should be noted. First, the Malik and Malik (2011) twelve-step guide was originally developed for integration in medical school curricula and, to our knowledge, has not been explicitly applied outside of that context. While prior work (Costley, 2015) suggests its adaptability for secondary and elementary education, the present study represents a novel application for a family-based curriculum. Future research should examine whether other integration approaches may provide a better fit for family engagement curricula. Next, while the curriculum in the present study was developed by experts and is grounded in theory, its effectiveness in increasing physical activity or other health behaviors in children and families has not been tested. Additionally, only one partner organization (MiSHCA) has been explicitly surveyed regarding the value of the curriculum. It would be helpful to have the perspective of community members and other organizations quantified as well. Finally, although many module authors are parents and/or work closely with families, end-users (parents and families) were not directly involved in its creation, potentially missing their valuable perspective.

## 5 Conclusion

The COVID-19 pandemic highlighted the breadth of challenges children and families face, particularly as disruptions in daily life exacerbated inequities and limited access to resources needed to meet both basic and developmental needs. Using Maslow’s hierarchy of needs as a guiding framework allowed us to organize these diverse needs systematically and to design a curriculum that could address them across all levels of the hierarchy.

Through a systematic development process, we created a 12-module family toolkit that integrates physical activity as a unifying mechanism to promote health and well-being. Each module is designed to help families practice health behaviors together at home, with the flexibility to tailor resources to their individual needs or progress sequentially to build knowledge, attitudes, and skills. By framing physical activity not only as a means to improve child health, but also as a strategy to support safety, belonging, esteem, and self-actualization, the toolkit directly responds to the disruptions and inequities intensified during the pandemic.

Finally, embedding this toolkit into the existing school health education curriculum offers an important opportunity to strengthen school-home connections, providing teachers with practical resources to share with parents. Future research will evaluate the effectiveness of this curriculum to: (1) improve family knowledge, attitudes, and skills to engage in health behaviors aligned with Maslow’s hierarchy of needs; (2) strengthen family relationships; and (3) increase physical activity participation and overall well-being among children.

## Data Availability

The raw data supporting the conclusions of this article will be made available by the authors, without undue reservation.
